# Adjunct Tendon Vibration and Bone Outcomes in Older Adults with Osteoporosis: A 12-Month Prospective Cohort Study

**DOI:** 10.3390/jcm15103798

**Published:** 2026-05-14

**Authors:** Konstantinos Moutaftsis, Aikaterini Anetaki, Constantine Anetakis, Eleftherios Panteris, Ioannis Chaniotakis, Ilias Pessach, Maria Chatzidimitriou, Petros Skepastianos, Eleni Andreadou, Mattheos Bobos, Paris Iakovidis, Thomas Apostolou, Stella Mitka

**Affiliations:** 1 Department of Physiotherapy, International Hellenic University, 57400 Thessaloniki, Greece; kostasmoutaftsis@gmail.com (K.M.); piakov@ihu.gr (P.I.); apostolouthomas@ihu.gr (T.A.); 2 Department of Biomedical Sciences, International Hellenic University, 57400 Thessaloniki, Greece; aikaanet@outlook.com (A.A.); kanetakis@bmsc.ihu.gr (C.A.); ioannischanio@gmail.com (I.C.); iliaspesach1980@gmail.com (I.P.); mchatzid952@gmail.com (M.C.); pskep@otenet.gr (P.S.); eandreadou@ihu.gr (E.A.); mbobos@icloud.com (M.B.); 3 Neonatology/Neonatal Intensive Care Unit, University General Hospital of Heraklion, University of Crete, 71110 Heraklion, Greece

**Keywords:** osteoporosis, bone mineral density, bone turnover markers, tendon vibration, older adults, cohort study

## Abstract

**Background/Objectives:** To evaluate the association between adjunct tendon vibration and changes over 12 months in dual-energy X-ray absorptiometry (DXA)-derived bone mineral density (BMD) T-score and bone turnover markers in older adults with osteoporosis receiving standard care in a non-randomised controlled cohort study. **Methods:** This 12-month prospective non-randomised controlled cohort study included 100 adults aged ≥60 years with DXA-confirmed osteoporosis recruited from orthopaedic clinics in the Greater Thessaloniki area. Fifty participants received adjunct tendon vibration therapy in addition to usual care, while 50 received usual care alone. Usual care consisted of calcium and vitamin D supplementation. The primary outcome was post-intervention BMD T-score, analysed using analysis of covariance (ANCOVA) adjusted for baseline T-score. Secondary outcomes included changes in bone turnover markers and calcium/phosphate metabolism. Sensitivity analysis was conducted using a linear mixed-effects model with repeated BMD measurements. **Results:** Baseline characteristics were comparable between groups. Over 12 months, the intervention group showed greater improvement in BMD T-score than controls (median change 0.90 [0.70–1.00] vs. −0.10 [−0.10–0.10], *p* < 0.001). The adjusted between-group difference was 0.871 (95% CI 0.773–0.968; *p* < 0.001). Results remained consistent after adjustment for age and sex. The mixed-effects model confirmed a significant group × time interaction (β = 0.922, 95% CI 0.806–1.038; *p* < 0.001). Bone resorption markers decreased more in the intervention group. The magnitude of the observed BMD improvement (~0.9 T-score units) is notable for a non-pharmacological intervention and should be interpreted cautiously. **Conclusions:** Adjunct tendon vibration was associated with a more favourable BMD trajectory and changes in bone turnover markers in older adults with osteoporosis receiving standard care. Given the non-randomised design and potential residual confounding, these findings should be interpreted as associative rather than causal.

## 1. Introduction

Osteoporosis is a major public health condition in older adults, characterized by reduced bone mineral density (BMD) and deterioration of bone microarchitecture, leading to increased skeletal fragility and fracture risk [1]. Age-related bone loss results from an imbalance between bone formation and resorption, primarily driven by reduced osteoblastic activity and relatively increased osteoclastic activity, resulting in progressive deterioration of skeletal integrity [2,3,4]. Fragility fractures, particularly of the hip and vertebrae, are associated with substantial morbidity, mortality, reduced quality of life, and increased healthcare burden [1,5].

Current management of osteoporosis is primarily based on pharmacological treatment. Antiresorptive agents, including bisphosphonates and denosumab, reduce fracture risk by inhibiting osteoclast-mediated bone resorption, while anabolic therapies such as teriparatide stimulate bone formation and improve skeletal strength [6,7,8,9]. However, long-term adherence to pharmacological therapy remains suboptimal due to treatment burden, adverse effects, comorbidities, and reduced tolerability in older populations [5,10,11]. Therefore, there is increasing interest in complementary non-pharmacological strategies that may support bone health in clinical practice. Mechanical loading is a key regulator of bone remodeling. Osteocytes act as mechanosensors that detect mechanical stimuli and convert them into biochemical signals regulating bone remodeling [2,3,12]. This mechanotransduction process mediates skeletal adaptation through intracellular signaling pathways. Similar mechanotransduction mechanisms have also been described in tendon tissue [12,13,14]. Reduced mechanical stimulation, as observed in aging, immobility, or sedentary behavior, is associated with increased bone resorption and progressive decline in BMD [10]. Among non-pharmacological approaches, whole-body vibration (WBV) has been investigated as a method to enhance skeletal loading. However, WBV has important limitations, including limited patient tolerability and discomfort during prolonged use. In addition, its effects on BMD remain inconsistent across clinical studies, with heterogeneous findings reported [15,16,17,18,19]. In addition, the translational gap between mechanistic evidence and consistent clinical benefit remains unresolved [18].

Targeted tendon vibration represents a more localized mechanical intervention. The rationale for local tendon vibration is supported by mechanobiological evidence showing that skeletal and tendon tissues respond to mechanical stimuli through mechanotransduction pathways [13,20]. Unlike whole-body vibration, it delivers localized oscillatory mechanical stimuli directly to muscle–tendon units, potentially inducing reflexive muscle activation and transmitting mechanical forces to adjacent skeletal structures. However, the exact biological mechanisms underlying its effects on bone remodelling in humans remain incompletely understood [3,12,17,18,21]. Preclinical evidence also suggests potential tendon–bone and neuromechanical crosstalk mechanisms that may contribute to skeletal adaptation [17,22,23].

Emerging clinical studies have investigated tendon vibration in older adults with osteoporosis, reporting improvements in bone mineral density and changes in bone turnover markers, with generally good tolerability [18,22,24,25]. In this context, bone turnover markers may provide early insight into skeletal response, as biochemical changes may precede measurable changes in BMD [25,26]. However, existing evidence remains limited by small sample sizes, short follow-up duration, and heterogeneity in intervention protocols. Given the multifactorial nature of osteoporosis, including impaired bone remodeling, reduced mechanical loading, hormonal changes, and age-related sarcopenia, the exploration of adjunctive non-pharmacological interventions is clinically relevant [4,24,27,28]. Targeted tendon vibration may represent a promising complementary strategy to standard care by enhancing mechanical signalling and supporting bone metabolism.

Importantly, in the present study, all participants received uniform standard care consisting of calcium and vitamin D supplementation, ensuring a consistent baseline treatment across groups [6]. However, information regarding previous exposure to anti-osteoporotic pharmacotherapy before study enrolment was not available and is acknowledged as a potential source of residual confounding.

Therefore, the present study aimed to evaluate whether adjunct local tendon vibration, applied in routine clinical practice, was associated with one-year changes in DXA-derived BMD T-score and selected bone turnover markers in older adults with osteoporosis. Given the non-randomised design, allocation based on clinical practice, and potential for residual confounding, the study was intended to provide real-world, exploratory, and hypothesis-generating evidence rather than to establish causal relationships.

## 2. Materials and Methods

**Study design and ethical approval**

This was a 12-month non-randomised prospective controlled cohort study evaluating the association between adjunct local tendon vibration and changes in bone mineral density (BMD) and biochemical markers of bone turnover in older adults with osteoporosis. The study was approved by the Research Ethics Committee of the International Hellenic University (Protocol No. 123/2025). All participants provided written informed consent. The study was conducted in accordance with the Declaration of Helsinki and applicable national regulations.

**Setting and participants**

Participants were recruited from orthopaedic clinics in the Greater Thessaloniki area and followed prospectively for 12 months. Eligible participants were adults aged ≥60 years with DXA-confirmed osteoporosis. In the analysed cohort, age ranged from 60 to 80 years. All participants received uniform standard osteoporosis care consisting of calcium (1000–1200 mg/day) and vitamin D (800–1000 IU/day). Exclusion criteria included malignancy, severe cardiovascular or neurological disease, chronic corticosteroid use, metallic implants or pacemakers, and use of anti-osteoporotic pharmacological agents (e.g., bisphosphonates, denosumab, or anabolic therapies), as well as other conditions considered likely to substantially affect bone metabolism. Information regarding prior exposure to anti-osteoporotic pharmacotherapy before enrolment was not available.

Group allocation was not randomised. The intervention group included 50 participants who had been prescribed adjunct tendon vibration therapy by their treating clinicians as part of routine strengthening and fall-prevention management, in addition to standard care. The control group included 50 comparable participants receiving standard care alone. Allocation was based on clinician judgment within routine clinical practice, without a predefined allocation algorithm. As treatment allocation was clinician-determined, the study is observational in nature.

**Sample size**

An a priori sample size calculation using G*Power (version 3.1) assumed a moderate effect size (Cohen’s d = 0.5), α = 0.05, and 80% power, yielding a minimum required sample of 86 participants. To account for potential attrition, 100 participants were enrolled. A moderate standardised effect size (Cohen’s d = 0.5) was selected a priori as a conservative and commonly used planning assumption for exploratory interventional studies when robust effect-size estimates from directly comparable local tendon-vibration studies are unavailable.

**Intervention Protocol (Tendon Vibration)**

The tendon vibration intervention consisted of a structured, supervised protocol delivered over a 12-month period. Participants in the intervention group completed three sessions per week, with each session lasting approximately 20–30 min, resulting in a total of approximately 144 sessions per participant throughout the study period. The intervention was delivered using a custom-built local tendon vibration device developed specifically for the purposes of this study, as no commercially available device met the required technical specifications. The system was engineered to generate controlled sinusoidal mechanical vibration with precise regulation of frequency and amplitude, ensuring high reproducibility of the applied mechanical stimulus across all sessions.

Prior to study initiation, the device was calibrated using a triaxial accelerometer (MPU6050), and calibration stability was periodically verified at predefined intervals to ensure consistency of mechanical output over time. Following initial calibration, all stimulation parameters remained fixed and were not modified during the 12-month intervention period. Mechanical vibration was delivered via a rigid applicator head designed to ensure stable and reproducible contact with the target tendon. The applicator was positioned perpendicular to the tendon surface with light, standardized, and manually controlled (non-compressive) contact pressure, allowing efficient transmission of mechanical vibration while avoiding compression of surrounding soft tissues. All contact conditions were standardized across participants through trained manual positioning.

All interventions were administered by the same trained physiotherapist, following a strictly standardized operating protocol. The operator received structured training prior to study initiation to ensure uniformity in participant positioning, applicator placement, contact pressure, and session timing, thereby minimizing inter-operator variability. The vibration stimulus was delivered using fixed parameters: frequency 100 Hz (±2 Hz), amplitude 0.3–0.7 mm, sinusoidal waveform, and a duty cycle of 10 s ON/10 s OFF, with inter-session variability < 5%. These parameters remained constant throughout the study period.

Vibration was applied bilaterally to the Achilles and quadriceps (patellar) tendons. Participants were positioned in standardized postures (seated for patellar tendon application and supine or semi-recumbent for Achilles tendon application). Each session included alternating stimulation between the two anatomical sites with equal distribution of exposure time. All sessions were fully supervised. Adherence was monitored using attendance logs and participant diaries, demonstrating overall compliance exceeding 90%.

**DXA assessment**

Bone mineral density was assessed at baseline and after 12 months using dual-energy X-ray absorptiometry (DXA; Hologic Inc., Bedford, MA, USA) performed by the same certified technologist. Measurements included the lumbar spine and proximal femur as part of routine clinical evaluation, with a reported coefficient of variation < 1%. Site-specific BMD values (g/cm^2^) and regional T-scores were available in the dataset. The primary outcome was defined as the T-score from the anatomical site with the lowest value at baseline, selected from the lumbar spine and proximal femur, in accordance with established diagnostic criteria for osteoporosis and the official positions of the International Society for Clinical Densitometry (ISCD), as well as recent updates on DXA interpretation and monitoring in clinical practice [29,30]. The same anatomical site identified at baseline was subsequently used for longitudinal comparison at 12 months to ensure consistency over time. Post-intervention BMD T-score was selected as the primary outcome because the available sample size and follow-up duration were insufficient to support reliable analysis of fracture or hospitalisation endpoints, whereas BMD is an established intermediate skeletal outcome that is sensitive to change over 12 months.

**Biochemical measurements**

Fasting blood samples were collected at baseline and at 12 months and analysed using validated automated and immunoassay methods according to manufacturer instructions. Bone formation markers included osteocalcin (OC), bone-specific alkaline phosphatase (b-ALP), procollagen type I N-terminal propeptide (P1NP), and total alkaline phosphatase (t-ALP). Bone resorption markers included N-terminal telopeptide of type I collagen (NTX), C-terminal telopeptide of type I collagen (CTX), and tartrate-resistant acid phosphatase (TRAP). Mineral and hormonal parameters included calcium (Ca), phosphate (P), parathyroid hormone (PTH), and 25-hydroxyvitamin D [25(OH)D].

**Safety reporting and Adverse Event Monitoring**

No adverse events leading to study discontinuation were recorded during the study period. Safety monitoring was conducted prospectively using a predefined, standardised protocol with systematic assessment at each study visit. Adverse events were captured using a structured questionnaire, and the frequency of recorded events in each group is summarised in Supplementary Table S1. The questionnaire assessed falls, fractures, musculoskeletal and neurological symptoms, and device-related events, and included predefined categories for event severity (mild, moderate, severe) and the assessor’s judgement regarding relatedness to the intervention. In addition, participant reports between scheduled visits were used to document any interim adverse events occurring outside routine assessments.

**Statistical analysis**

Participants were assessed at baseline and after 12 months. Statistical analysis was performed in the Python 3.11.2 environment, using pandas 2.2.3 for data management and reshaping (wide-to-long and long-to-wide formats), SciPy 1.14.1 for conventional statistical tests (including the Shapiro–Wilk test, *t*-tests, Mann–Whitney U test, Wilcoxon signed-rank test, and Spearman correlation), and statsmodels 0.14.3 for fitting linear models (OLS/ANCOVA) and estimating the corresponding confidence intervals.

Normality of continuous variables was evaluated using the Shapiro–Wilk test. Continuous data are presented as mean ± standard deviation (SD) or median (interquartile range, IQR), as appropriate.

Baseline between-group comparisons were performed using Welch’s *t*-test or the Mann–Whitney U test depending on data distribution. Within-group changes over time were assessed using paired *t*-tests or Wilcoxon signed-rank tests, as appropriate. Between-group comparisons of change scores were conducted using Welch’s *t*-test or Mann–Whitney U test.

No missing data were identified for the variables included in the primary analysis; therefore, complete-case analysis was used and no imputation procedures were required.

The primary confirmatory analysis was performed using analysis of covariance (ANCOVA), with post-intervention BMD T-score as the dependent variable, study group as the independent variable, and baseline BMD T-score included as a covariate. ANCOVA assumptions were formally assessed, including homogeneity of regression slopes by testing the group × baseline BMD T-score interaction, inspection of residual-versus-fitted and normal Q–Q plots, and evaluation of influential observations using standard influence diagnostics. Additional multivariable linear regression models were used to assess the robustness of the group effect after adjustment for age, sex, baseline 25(OH)D, and change in 25(OH)D (Δ25[OH]D).

An exploratory interaction model including a group × baseline BMD T-score term was fitted to examine whether treatment effects differed according to baseline skeletal status. As a sensitivity analysis, a linear mixed-effects model with a subject-specific random intercept was applied to repeated BMD T-score measurements, with fixed effects for group, time, and the group × time interaction. The model was estimated using maximum likelihood. Exploratory analyses included Spearman correlation analyses between changes in BMD T-score and changes in biochemical markers. In addition, separate multivariable linear regression models were constructed to examine associations between change in BMD T-score and each biomarker, adjusting for group, age, baseline BMD T-score, and Δ25(OH)D. Given the number of secondary and exploratory analyses and the absence of adjustment for multiple comparisons, these results should be interpreted as hypothesis-generating and descriptive. Statistical significance for the primary outcome was defined as a two-sided *p*-value < 0.05.

## 3. Results

A total of 100 participants were included in the analytic cohort, with 50 individuals in each group (16 men and 34 women per group), all Greek nationals. Repeated BMD T-score measurements at baseline and 12 months were available for all participants, yielding 200 observations for the mixed-effects analysis. No missing BMD data were identified.

At baseline, the intervention and control groups were comparable across demographic, densitometric, and biochemical parameters (Table 1). Median age was 69.0 (67.0–72.0) years in the intervention group and 69.5 (68.0–72.8) years in the control group (*p* = 0.280). Baseline BMD T-score was −3.80 (−4.00 to −3.60) in the intervention group and −3.80 (−3.90 to −3.50) in the control group (*p* = 0.166). No statistically significant between-group differences were observed for bone turnover markers or calcium–phosphate metabolism variables.

Over the 12-month follow-up, the intervention group demonstrated a marked improvement in BMD T-score, with a median increase of 0.90 (0.70 to 1.00), corresponding to an approximate relative improvement of 24% from baseline values. In contrast, the control group showed minimal change (−0.10 [−0.10 to 0.10]) (Table 2 and Table 3). This magnitude of change is notable for a non-pharmacological intervention and is therefore interpreted cautiously in light of the study’s non-randomised design. Consistent with the densitometric findings, bone resorption markers (NTX, CTX, and TRAP) decreased substantially in the intervention group, whereas they remained stable or increased in the control group. P1NP increased in both groups but to a greater extent in the intervention group (+55.00 vs. +24.85 ng/mL, *p* < 0.001), suggesting a more pronounced bone formation response. Other biochemical parameters, including calcium, phosphate, and PTH remained broadly stable, while 25(OH)D increased modestly in the intervention group.

Between-group comparisons of change scores confirmed a significantly more favourable one-year BMD T-score trajectory in the intervention group (*p* < 0.001), along with significant differences in key bone turnover markers, including bone alkaline phosphatase, P1NP, NTX, CTX, and TRAP (Table 4). No statistically significant differences were observed for osteocalcin, total alkaline phosphatase, calcium, phosphate, or PTH.

In the primary ANCOVA model, the adjusted difference in post-intervention BMD T-score between groups was 0.871 units (95% CI 0.773 to 0.968; *p* < 0.001) after adjustment for baseline BMD T-score (Table 5). This estimate remained stable across multivariable models including age, sex, baseline 25(OH)D, and Δ25(OH)D (range 0.864–0.886). Baseline BMD T-score was the strongest independent predictor of outcome, whereas age, sex, and vitamin D-related variables were not consistently associated with post-intervention BMD.

Exploratory analysis suggested a potential interaction between baseline skeletal status and treatment response, with greater improvements observed in participants with lower baseline BMD T-scores. However, given the post hoc nature of this analysis and the non-randomised design, these findings should be interpreted with caution (Supplementary Table S2).

Correlation analyses indicated that improvements in BMD T-score were positively associated with increases in P1NP and inversely associated with changes in NTX, CTX, and TRAP (Supplementary Tables S2 and S3). However, after adjustment for group allocation, age, baseline BMD T-score, and change in 25(OH)D, most associations were attenuated and no longer statistically significant. In adjusted models, change in t-ALP remained inversely associated with BMD change (β = −0.016, 95% CI −0.030 to −0.002; *p* = 0.027), while change in Ca showed a positive association (β = 0.217, 95% CI 0.029 to 0.405; *p* = 0.024). The association with P1NP was of borderline significance (*p* = 0.073).

As a sensitivity analysis, the linear mixed-effects model demonstrated a significant group × time interaction (β = 0.922, 95% CI 0.806 to 1.038; *p* < 0.001), confirming a clear difference-in-differences pattern favouring of the intervention group (Table 6). Estimated marginal means indicated that BMD T-score remained essentially unchanged in the control group, whereas it improved from −3.828 to −2.936 over 12 months in the intervention group.

Additional details regarding safety monitoring and exploratory analyses are provided in the Supplementary Materials, including the exploratory ANCOVA interaction model (Supplementary Table S2), correlation analyses (Supplementary Table S3), adjusted regression models (Supplementary Tables S4 and S5), and estimated marginal means from the mixed-effects model (Supplementary Table S6).

## 4. Discussion

In this prospective non-randomised controlled cohort study, adjunct tendon vibration was associated with a more favourable 12-month trajectory in DXA-derived BMD T-score compared with standard care consisting of calcium and vitamin D supplementation. This association was consistent across unadjusted analyses, the primary ANCOVA model, and sensitivity analyses using linear mixed-effects modelling, indicating a stable relationship between group allocation and longitudinal changes in bone mineral density. Osteoporosis is a multifactorial skeletal disorder characterised by impaired bone strength and increased fracture risk, providing the clinical context for interpreting such changes in BMD [1,2]. However, given the non-randomised design, these findings should be interpreted as associative rather than causal. The magnitude of the observed effect, while clinically relevant, should also be interpreted with caution in the context of potential residual confounding and the absence of randomisation [10,17,22], particularly as the observed improvement appears greater than typically reported for non-pharmacological interventions, raising the possibility of unmeasured contributing factors.

These findings are broadly consistent with previous studies investigating vibration-based interventions, including whole-body vibration protocols, which have reported modest improvements in BMD and bone turnover markers, albeit with substantial heterogeneity in study design, exposure parameters, and duration [15,16,17,18,31]. Compared with these approaches, the magnitude of change observed in the present study appears relatively pronounced, which may relate to the targeted nature of the intervention, the higher stimulation frequency, or cohort-specific characteristics, although this cannot be definitively established. In this context, the present study extends existing evidence by providing prospective 12-month data using a localized tendon-targeted mechanical stimulus, together with concurrent assessment of biochemical markers of bone turnover. This combined imaging–biochemical approach allows a more integrated evaluation of skeletal response compared with studies relying solely on densitometric outcomes.

Changes in bone turnover markers demonstrated a broadly coherent pattern, with reductions in bone resorption indices (CTX, NTX, TRAP) observed in the intervention group and a relatively greater increase in the bone formation marker P1NP compared with controls. Nevertheless, the response was not uniform across all formation-related markers, as osteocalcin and total alkaline phosphatase did not show consistent or statistically significant changes. Accordingly, these biochemical findings should be interpreted as supportive indicators of altered bone remodelling dynamics rather than as evidence of a specific or isolated biological pathway, given the known biological variability and context-dependence of bone turnover markers [25,26].

From a mechanistic standpoint, the observed findings are consistent with established principles of skeletal mechanotransduction, whereby mechanical stimuli are transduced into biochemical and cellular signals regulating osteocyte activity and downstream bone remodelling pathways. This may involve coordinated modulation of mechanosensitive pathways such as Wnt/β-catenin signalling and the RANKL/osteoprotegerin axis, although these were not directly assessed in the present study [3,12,21,32,33]. In addition, emerging evidence on tendon–bone and neuromuscular–skeletal crosstalk suggests that localized mechanical stimulation may exert systemic effects through integrated biomechanical and biochemical pathways, although the precise contribution of these pathways to systemic skeletal adaptation remains to be fully elucidated [34,35,36].

From a clinical perspective, local tendon vibration may represent a practical and low-burden adjunct non-pharmacological intervention, particularly in older adults with limited exercise tolerance or mobility constraints, where conventional loading-based strategies are difficult to implement [6,37]. This may be particularly relevant for frail individuals or those at high fall risk, in whom adherence to exercise-based interventions is often limited. Importantly, all participants received standard care with calcium and vitamin D supplementation, ensuring a uniform baseline supportive treatment across groups and strengthening internal comparability.

No adverse events leading to discontinuation of the intervention were recorded during the study period. Safety monitoring was conducted using a structured approach to adverse event assessment, based on a predefined list of potential events and systematic clinical evaluation at each participant contact. Adverse events were actively assessed through structured interviews conducted under the supervision of a clinical investigator, who was a specialised physiotherapist and performed all safety evaluations throughout the study period. These assessments were supplemented by participant reports between study visits. Therefore, safety data were collected prospectively and in a standardised manner. Furthermore, adherence to the vibration protocol was not systematically quantified beyond attendance at treatment sessions, and potential variations in compliance may have influenced the observed outcomes.

A key methodological consideration is the definition of the primary bone outcome as a single derived T-score, calculated as the lowest value between lumbar spine and femoral neck measurements. While this approach increases diagnostic sensitivity for osteoporosis classification, it limits anatomical specificity and precludes site-specific interpretation of skeletal responses [21,33].

The non-randomised design represents an important limitation, introducing the possibility of residual confounding due to unmeasured variables such as functional status, physical activity levels, fall risk, and frailty-related factors. Although multivariable adjustment was performed for key covariates, residual bias cannot be excluded [10,17,22]. In addition, group allocation based on clinician judgement may have introduced selection bias, potentially favouring individuals with differing baseline risk profiles or health status.

The study included both men and women; however, it was not powered to detect sex-specific differences, and potential sex-related variability in skeletal response cannot be excluded, particularly given known differences in bone metabolism, hormonal status, and response to mechanical loading between sexes [10,37].

Because participants were recruited from orthopaedic clinics within a single metropolitan region under routine clinical practice, some site-related variation in referral patterns and clinical decision-making cannot be excluded.

In addition, multiple exploratory analyses were conducted without formal adjustment for multiplicity; therefore, secondary and exploratory findings should be interpreted as descriptive and hypothesis-generating [16,37].

Taken together, the present findings support the concept that targeted mechanical stimulation may modulate skeletal homeostasis in a clinically meaningful manner; however, confirmation in rigorously designed studies is required.

Finally, future research should prioritise randomized controlled designs, incorporation of site-specific and absolute BMD measurements (g/cm^2^), and comprehensive assessment of clinically relevant endpoints, including fracture incidence and functional outcomes. Such studies will be essential to establish the clinical effectiveness and translational relevance of tendon vibration as a potential adjunct strategy in osteoporosis management [16,31,37].

Emerging biomarkers, including the RANKL/osteoprotegerin system, sclerostin, periostin, and fibroblast growth factor-23 (FGF-23), together with multi-omic and extracellular vesicle–based approaches, may further enhance mechanistic understanding of bone remodelling processes [12,25,26,32]. However, these remain largely investigational, and the present study relied exclusively on established biochemical markers of bone turnover [25,26]. Consequently, the observed changes should be interpreted within the constraints of these conventional but relatively non-specific indicators of skeletal metabolism.

## 5. Conclusions

In this non-randomised prospective controlled cohort study, adjunct local tendon vibration was associated with a significantly more favourable one-year DXA-derived BMD T-score trajectory and concurrent patterns of change in bone turnover markers in older adults with osteoporosis receiving uniform standard care with calcium and vitamin D supplementation.

Given the observational design and clinician-determined treatment allocation, causal inference cannot be established, and residual confounding from unmeasured variables such as physical activity, frailty status, and functional capacity cannot be excluded. In addition, potential selection bias related to non-randomised group allocation may have influenced the observed associations. Nevertheless, the consistency of the findings across ANCOVA and linear mixed-effects modelling, together with concordant patterns in bone turnover markers, supports the robustness of the observed association.

Importantly, these results do not establish efficacy but provide clinically relevant real-world evidence suggesting that tendon vibration may be a promising adjunct mechanical intervention in osteoporosis management. The intervention demonstrated feasibility, good tolerability, and measurable skeletal associations over a clinically meaningful follow-up period. However, the magnitude of the observed effect should be interpreted with caution, as it appears greater than typically reported for non-pharmacological interventions and may partly reflect unmeasured confounding or cohort-specific characteristics. The study included both men and women but was not powered to detect sex-specific differences; therefore, potential sex-related variability in response cannot be excluded.

Future research should prioritise adequately powered randomized controlled trials with standardized pharmacological documentation, site-specific and absolute BMD measurements (g/cm^2^), and clinically meaningful endpoints including fracture incidence, falls risk, and functional outcomes. Such studies are essential to determine whether the observed associations translate into clinically meaningful benefit and to define the potential role of tendon vibration within evidence-based osteoporosis care pathways.

## Figures and Tables

**Table 1:** Baseline demographic and laboratory characteristics by study group.

Variable	Intervention Group	Control Group	*p*-Value
**Age (years)**	69.00 (67.00 to 72.00)	69.50 (68.00 to 72.75)	0.280
**Baseline BMD T-score**	−3.80 (−4.00 to −3.60)	−3.80 (−3.90 to −3.50)	0.166
**Osteocalcin at baseline (ng/mL)**	9.10 (7.80 to 11.20)	9.15 (7.83 to 11.15)	0.671
**Total alkaline phosphatase at baseline (U/L)**	62.00 (56.00 to 66.00)	63.00 (53.75 to 66.00)	0.986
**Bone alkaline phosphatase at baseline (U/L)**	36.10 (33.30 to 42.00)	35.65 (33.30 to 42.00)	0.970
**P1NP at baseline (ng/mL)**	59.70 (38.20 to 78.17)	59.70 (38.20 to 78.17)	1.000
**NTX at baseline (nM BCE)**	22.40 (22.10 to 28.40)	22.40 (22.10 to 28.40)	1.000
**CTX at baseline (ng/mL)**	1.42 (1.32 to 1.68)	1.42 (1.29 to 1.62)	0.510
**TRAP at baseline (ng/mL)**	9.20 (8.10 to 12.22)	9.20 (8.10 to 12.28)	0.997
**Calcium at baseline (mg/dL)**	8.82 ± 0.27	8.82 ± 0.28	0.943
**Phosphate at baseline (mg/dL)**	4.00 (3.90 to 4.50)	4.00 (3.90 to 4.50)	0.227
**PTH at baseline (pg/mL)**	31.00 (22.00 to 48.50)	31.00 (19.75 to 41.75)	0.315
**25(OH)D at baseline (ng/mL)**	17.00 (14.00 to 21.00)	17.50 (14.00 to 21.00)	0.822

Data are presented as median (interquartile range, IQR) for non-normally distributed variables and as mean ± SD for normally distributed variables. *p*-values are based on the Mann–Whitney U test or Welch’s *t*-test, as appropriate.

**Table 2:** Within-group changes over 12 months in the intervention group.

Variable	Baseline	12 Months	Change (Δ)	*p*-Value
**BMD T-score**	−3.80 (−4.00 to −3.60)	−3.00 (−3.10 to −2.80)	0.90 (0.70 to 1.00)	<0.001
**Osteocalcin (ng/mL)**	9.10 (7.80 to 11.20)	8.00 (7.30 to 11.20)	−0.35 (−1.00 to 0.20)	0.052
**Total alkaline phosphatase (U/L)**	62.00 (56.00 to 66.00)	60.50 (56.00 to 65.75)	−1.00 (−4.00 to 3.00)	0.526
**Bone alkaline phosphatase (U/L)**	36.10 (33.30 to 42.00)	31.80 (28.95 to 36.08)	−4.60 (−7.05 to −3.00)	<0.001
**P1NP (ng/mL)**	59.70 (38.20 to 78.17)	123.20 (98.88 to 138.20)	55.00 (40.00 to 87.50)	<0.001
**NTX (nM BCE)**	22.40 (22.10 to 28.40)	16.70 (14.82 to 17.82)	−8.55 (−10.60 to −5.55)	<0.001
**CTX (ng/mL)**	1.42 (1.32 to 1.68)	0.91 (0.68 to 0.99)	−0.70 (−0.98 to −0.41)	<0.001
**TRAP (ng/mL)**	9.20 (8.10 to 12.22)	5.45 (4.83 to 6.20)	−4.70 (−6.08 to −3.00)	<0.001
**Calcium (mg/dL)**	8.82 ± 0.27	8.85 ± 0.24	0.03 ± 0.40	0.621
**Phosphate (mg/dL)**	4.00 (3.90 to 4.50)	4.05 (3.90 to 4.50)	0.00 (−0.10 to 0.45)	0.096
**PTH (pg/mL)**	31.00 (22.00 to 48.50)	31.00 (24.25 to 40.50)	−3.50 (−10.00 to 9.75)	0.069
**25(OH)D (ng/mL)**	17.00 (14.00 to 21.00)	19.00 (17.00 to 24.00)	3.00 (1.00 to 3.00)	<0.001

Change scores are expressed as post-intervention minus pre-intervention values [Δ = after − before]. *p*-values are paired within-group comparisons derived from the paired *t*-test or Wilcoxon signed-rank test, as appropriate.

**Table 3:** Within-group changes over 12 months in the control group.

Variable	Baseline	12 Months	Change (Δ)	*p*-Value
**BMD T-score**	−3.80 (−3.90 to −3.50)	−3.80 (−4.00 to −3.50)	−0.10 (−0.10 to 0.10)	0.130
**Osteocalcin (ng/mL)**	9.15 (7.83 to 11.15)	9.15 (8.12 to 10.95)	0.00 (−1.02 to 0.95)	0.783
**Total alkaline phosphatase (U/L)**	63.00 (53.75 to 66.00)	63.00 (53.75 to 66.00)	0.00 (0.00 to 0.00)	1.000
**Bone alkaline phosphatase (U/L)**	35.65 (33.30 to 42.00)	35.65 (33.30 to 42.00)	−1.55 (−4.75 to 5.08)	1.000
**P1NP (ng/mL)**	59.70 (38.20 to 78.17)	92.70 (80.58 to 98.42)	24.85 (10.05 to 49.45)	<0.001
**NTX (nM BCE)**	22.40 (22.10 to 28.40)	28.10 (25.47 to 29.30)	2.50 (1.00 to 4.80)	<0.001
**CTX (ng/mL)**	1.42 (1.29 to 1.62)	1.52 (1.39 to 1.75)	0.10 (0.04 to 0.10)	<0.001
**TRAP (ng/mL)**	9.20 (8.10 to 12.28)	11.90 (9.22 to 13.20)	0.90 (0.00 to 1.48)	0.011
**Calcium (mg/dL)**	8.82 ± 0.28	8.82 ± 0.28	0.00 ± 0.00	1.000
**Phosphate (mg/dL)**	4.00 (3.90 to 4.50)	4.00 (3.90 to 4.50)	0.00 (−0.30 to 0.30)	0.938
**PTH (pg/mL)**	31.00 (19.75 to 41.75)	29.00 (18.25 to 33.50)	0.00 (−4.00 to 0.00)	0.101
**25(OH)D (ng/mL)**	17.50 (14.00 to 21.00)	17.50 (14.00 to 21.00)	0.50 (−5.00 to 4.00)	0.649

Change scores are expressed as post-intervention minus pre-intervention values [Δ = after − before]. *p*-values are paired within-group comparisons derived from the paired *t*-test or Wilcoxon signed-rank test, as appropriate.

**Table 4:** Between-group comparison of one-year change scores.

Variable	Δ Intervention Group	Δ Control Group	*p*-Value
**BMD T-score**	0.90 (0.70 to 1.00)	−0.10 (−0.10 to 0.10)	<0.001
**Osteocalcin (ng/mL)**	−0.35 (−1.00 to 0.20)	0.00 (−1.02 to 0.95)	0.256
**Total alkaline phosphatase (U/L)**	−1.00 (−4.00 to 3.00)	0.00 (0.00 to 0.00)	0.356
**Bone alkaline phosphatase (U/L)**	−4.60 (−7.05 to −3.00)	−1.55 (−4.75 to 5.08)	<0.001
**P1NP (ng/mL)**	55.00 (40.00 to 87.50)	24.85 (10.05 to 49.45)	<0.001
**NTX (nM BCE)**	−8.55 (−10.60 to −5.55)	2.50 (1.00 to 4.80)	<0.001
**CTX (ng/mL)**	−0.70 (−0.98 to −0.41)	0.10 (0.04 to 0.10)	<0.001
**TRAP (ng/mL)**	−4.70 (−6.08 to −3.00)	0.90 (0.00 to 1.48)	<0.001
**Calcium (mg/dL)**	0.03 ± 0.40	0.00 ± 0.00	0.621
**Phosphate (mg/dL)**	0.00 (−0.10 to 0.45)	0.00 (−0.30 to 0.30)	0.335
**PTH (pg/mL)**	−3.50 (−10.00 to 9.75)	0.00 (−4.00 to 0.00)	0.639
**25(OH)D (ng/mL)**	3.00 (1.00 to 3.00)	0.50 (−5.00 to 4.00)	0.006

Change scores were calculated as post-intervention minus pre-intervention values [Δ = after − before]. Calcium was compared using Welch’s *t*-test; all other variables were compared using the Mann–Whitney U test.

**Table 5:** Primary ANCOVA and multivariable linear models for post-intervention BMD T-score.

Model	Group Effect β (95% CI)	*p*-Value	Baseline BMD β (*p*-Value)	Age β (*p*-Value)	Sex β (*p*-Value)	Baseline 25(OH)D β (*p*-Value)	Δ25(OH)D β (*p*-Value)	Adjusted R^2^
**Primary ANCOVA: group + baseline BMD**	0.871 (0.773 to 0.968)	<0.001	0.507 (<0.001)	–	–	–	–	0.738
**+age**	0.874 (0.774 to 0.973)	<0.001	0.504 (<0.001)	0.004 (0.582)	–	–	–	0.737
**+age + sex**	0.879 (0.781 to 0.976)	<0.001	0.501 (<0.001)	0.011 (0.343)	−0.106 (0.310)	–	–	0.740
**+age + baseline 25(OH)D**	0.872 (0.773 to 0.972)	<0.001	0.470 (<0.001)	0.004 (0.558)	–	0.012 (0.095)	–	0.747
**+age + Δ25(OH)D**	0.886 (0.785 to 0.986)	<0.001	0.504 (<0.001)	0.004 (0.579)	–	–	−0.004 (0.179)	0.736
**+age + baseline 25(OH)D + Δ25(OH)D**	0.864 (0.767 to 0.961)	<0.001	0.465 (<0.001)	0.004 (0.560)	–	0.014 (0.156)	0.003 (0.613)	0.745

The group effect β represents the adjusted difference between the intervention and control groups in post-intervention BMD T-score. Sex was coded as male versus female, with female as the reference category. CI, confidence interval; Δ, change over follow-up (Supplementary Tables S3–S5 have all the details for coefficients and estimate marginal means for all models created).

**Table 6:** Linear mixed-effects model for repeated BMD T-score measurements.

Parameter	β Estimate	SE	z	*p*-Value	95% CI
**Intercept (control group, baseline)**	−3.724	0.042	−87.992	<0.001	−3.807 to −3.641
**Intervention group (vs control) at baseline**	−0.104	0.060	−1.738	0.082	−0.221 to 0.013
**Time: 12 months (vs baseline) in control group**	−0.030	0.042	−0.717	0.474	−0.112 to 0.052
**Group × time interaction**	0.922	0.059	15.574	<0.001	0.806 to 1.038

A linear mixed-effects model with a subject-specific random intercept was fitted with fixed effects for group, time, and the group × time interaction. Estimates were obtained by maximum likelihood. The model included 200 observations from 100 participants. Log-likelihood = −27.381; AIC = 66.761; BIC = 86.551.

## Data Availability

The data are not openly available due to reasons of sensitivity and are available from the corresponding author upon reasonable request.

## Supplementary Materials

The following supporting information can be downloaded at: https://www.mdpi.com/article/10.3390/jcm15103798/s1, Supplementary Table S1. Adverse events during the study period; Supplementary Table S2: Exploratory ANCOVA model with group × baseline BMD T-score interaction and adjusted contrasts; Supplementary Table S3: Spearman correlations between change in BMD T-score and changes in biochemical markers; Supplementary Table S4: Adjusted linear regression models for change in BMD T-score according to changes in biochemical markers; Supplementary Table S5: Detailed coefficient estimates from selected adjusted regression models for change in BMD T-score; Supplementary Table S6: Estimated marginal means for BMD T-score by group and time point from the mixed-effects model.

## Abbreviations

The following abbreviations are used in this manuscript:

Abbreviation	Full term
BMD	Bone Mineral Density
DXA	Dual-Energy X-ray Absorptiometry
OC	Osteocalcin
t-ALP	Total Alkaline Phosphatase
b-ALP	Bone-Specific Alkaline Phosphatase
P1NP	Procollagen Type 1 N-Terminal Propeptide
CTX	C-Terminal Telopeptide
NTX	N-Terminal Telopeptide
TRAP	Tartrate-Resistant Acid Phosphatase
PTH	Parathyroid Hormone
25(OH)D	25-Hydroxyvitamin D
P	Phosphate
Ca	Calcium
