# Supplementary material for: Adjunct Tendon Vibration and Bone Outcomes in Older Adults with Osteoporosis: A 12-Month Prospective Cohort Study
Source: J Clin Med. 2026 May 14;15(10):3798. doi: 10.3390/jcm15103798 (PMC13206889; doi:10.3390/jcm15103798)
Supplement: Supplementary file 1 [file jcm-15-03798-s001.zip › jcm-4302797-supplementary.pdf]

SUPPLEMENTARY MATERIALS

Supplementary Table S1. Adverse events during the study period.

Adverse event	Tendon vibration group (n = 50)	Control group (n = 50)
**Falls**	5 (10%)	6 (12%)
**Fractures**	0 (0%)	0 (0%)
**Muscle pain**	10 (20%)	11 (22%)
**Local discomfort (device-related)**	4 (8%)	0 (0%)
**Dizziness / feeling of instability**	2 (4%)	3 (6%)
**Other adverse events**	1 (2%)	1 (2%)

Abbreviations: n, number of participants experiencing at least one event in each category. Data are presented as number (percentage). The control group did not receive tendon vibration; therefore, device-related adverse events were not expected in this group.

Adverse events were prospectively collected using a standardized structured questionnaire administered by a single assessor at each study visit. Severity and relatedness to the intervention were pre-specified and systematically evaluated.

Adverse Event Questionnaire

Study: ___________________________
Participant ID: ___________________
Date: ____ / ____ / ______
Assessor (single investigator): _________________________

Note

This questionnaire is a structured tool for recording adverse events and is completed at each study visit through a clinical interview conducted by the same single assessor.

1. General safety assessment

Since the previous visit, did any adverse event occur?

☐ No
☐ Yes (please complete below)

2. Falls

Did you experience any falls?

☐ No
☐ Yes → number of falls: _______

• Was there any injury? ☐ No ☐ Yes (describe: ____________)
• Severity: ☐ Mild ☐ Severe
• Outcome: ☐ Full recovery ☐ Persistent ☐ Other: ______

3. Fractures

Did a fracture occur?

☐ No
☐ Yes

• Location: __________________________
• Imaging confirmation: ☐ Yes ☐ No
• Severity: ☐ Severe
• Outcome: ☐ Treated / managed ☐ Other: ______

4. Musculoskeletal symptoms

• Muscle pain: ☐ No ☐ Yes
• Local discomfort (device-related): ☐ No ☐ Yes
• Intensity: ☐ Mild ☐ Moderate ☐ Severe
• Outcome: ☐ Transient ☐ Persistent

5. Neurological / systemic symptoms

• Dizziness: ☐ No ☐ Yes
• Feeling of instability: ☐ No ☐ Yes
• Other symptoms: ______________________
• Intensity: ☐ Mild ☐ Moderate ☐ Severe
• Outcome: ☐ Transient ☐ Persistent

6. Relationship to intervention

In the assessor's judgment, is the event related to the intervention?

☐ Not related
☐ Unlikely related
☐ Possibly related
☐ Definitely related

7. Severity of adverse event

☐ Mild (no intervention required)
☐ Moderate (medical evaluation required)
☐ Severe (hospitalization or major intervention required)

8. Study discontinuation

Did the event lead to discontinuation from the study?

☐ No
☐ Yes

9. Investigator comments

………
………
…………………………………………………………………………

Supplementary Table S2. *Exploratory ANCOVA model with group × baseline BMD T-score interaction and adjusted contrasts.*

**Panel A. Interaction model**				
**Parameter**	β Estimate	95% CI lower	95% CI upper	p-value
**Intervention group (vs control)**	-1.878	-3.069	-0.687	0.002
**Baseline BMD T-score**	0.837	0.625	1.048	<0.001
**Group × baseline BMD T-score**	-0.727	-1.041	-0.413	<0.001
**Intercept**	-0.639	-1.429	0.152	0.112
**Panel B. Adjusted intervention–control difference at selected baseline BMD T-score values**				
**Baseline BMD T-score**	Adjusted difference	95% CI lower	95% CI upper	p-value
**-4.0**	1.030	0.912	1.148	<0.001
**-3.8**	0.885	0.789	0.980	<0.001
**-3.5**	0.667	0.537	0.796	<0.001

The interaction model was exploratory. Because baseline BMD T-score was not centred, the interaction contrasts are more clinically interpretable than the main group coefficient in isolation.

Supplementary Table S3. *Spearman correlations between change in BMD T-score and changes in biochemical markers.*

**Change in marker**	**Spearman's ϱ**	**95% CI**	**p-value**
**Osteocalcin**	-0.146	-0.332 to 0.052	0.148
**Total alkaline phosphatase**	-0.209	-0.390 to -0.013	0.037
**Bone alkaline phosphatase**	-0.268	-0.441 to -0.075	0.007
**P1NP**	0.432	0.257 to 0.579	<0.001
**NTX**	-0.686	-0.778 to -0.566	<0.001
**CTX**	-0.736	-0.815 to -0.631	<0.001
**TRAP**	-0.732	-0.812 to -0.625	<0.001
**Calcium**	0.199	0.002 to 0.380	0.048
**Phosphate**	-0.268	-0.441 to -0.075	0.007
**PTH**	-0.093	-0.284 to 0.106	0.359
**25(OH)D**	0.194	-0.003 to 0.376	0.054

Correlations were exploratory and unadjusted. Confidence intervals are 95% confidence intervals. N = 100.

Supplementary Table S4. *Adjusted linear regression models for change in BMD T-score according to changes in biochemical markers.*

**Change in marker**	**β (95% CI)**	**p-value**
**Osteocalcin**	-0.008 (-0.034 to 0.019)	0.575
**Total alkaline phosphatase**	-0.016 (-0.030 to -0.002)	0.027
**Bone alkaline phosphatase**	0.001 (-0.005 to 0.007)	0.704
**P1NP**	-0.002 (-0.004 to 0.000)	0.073
**NTX**	-0.001 (-0.027 to 0.026)	0.966
**CTX**	0.042 (-0.163 to 0.248)	0.686
**TRAP**	-0.006 (-0.027 to 0.015)	0.585
**Calcium**	0.217 (0.029 to 0.405)	0.024
**Phosphate**	0.007 (-0.121 to 0.134)	0.918
**PTH**	-0.003 (-0.007 to 0.001)	0.178

Each row represents a separate model with change in BMD T-score as the dependent variable. All models were adjusted for group, age, baseline BMD T-score, and Δ25(OH)D. These analyses were exploratory and p-values should be interpreted descriptively.

Supplementary Table S5. *Detailed coefficient estimates from selected adjusted regression models for change in BMD T-score.*

Predictor	Model Δt-ALP: β (95% CI)	p	Model ΔCa: β (95% CI)	p	Model ΔP1NP: β (95% CI)	p
**Intervention group (vs control)**	0.880 (0.784 to 0.977)	<0.001	0.882 (0.784 to 0.980)	<0.001	0.946 (0.817 to 1.075)	<0.001
**Baseline BMD T-score**	-0.485 (-0.709 to -0.261)	<0.001	-0.493 (-0.719 to -0.267)	<0.001	-0.534 (-0.788 to -0.279)	<0.001
**Age (years)**	0.005 (-0.009 to 0.020)	0.467	0.004 (-0.010 to 0.019)	0.548	0.004 (-0.010 to 0.018)	0.582
**Δ25(OH)D**	-0.004 (-0.010 to 0.001)	0.135	-0.005 (-0.011 to 0.001)	0.127	-0.005 (-0.011 to 0.001)	0.114
**Change in marker**	-0.016 (-0.030 to -0.002)	0.027	0.217 (0.029 to 0.405)	0.024	-0.002 (-0.004 to 0.000)	0.073

Each model included group, baseline BMD T-score, age, and Δ25(OH)D, differing only with respect to the biomarker-change term. The dependent variable in all models was change in BMD T-score.

Supplementary Table S6. *Estimated marginal means for BMD T-score by group and time point from the mixed-effects model.*

Group	Baseline	12 months	Change (12 months - baseline)
**Control group**	-3.724	-3.754	-0.030
**Intervention group**	-3.828	-2.936	0.892

Values are estimated marginal means derived from the mixed-effects model. The corresponding group × time interaction estimate was 0.922 T-score units.
